# Considering Maternal Dietary Modulators for Epigenetic Regulation and Programming of the Fetal Epigenome

**DOI:** 10.3390/nu7042748

**Published:** 2015-04-14

**Authors:** Abalo Chango, Igor P. Pogribny

**Affiliations:** 1 Polytechnic Institute LaSalle Beauvais, Department of Nutrition and Health Sciences, EGEAL UP:2012.10.101, F-60026 Beauvais Cedex, France; 2 Division of Biochemical Toxicology, Food and Drug Administration National Center for Toxicological Research, Jefferson, AR 72079, USA; E-Mail: igor.pogribny@fda.hhs.gov

**Keywords:** maternal diet, epigenetics, programming, DNA methylation, histone, microRNAs

## Abstract

Fetal life is characterized by a tremendous plasticity and ability to respond to various environmental and lifestyle factors, including maternal nutrition. Identification of the role of dietary factors that can modulate and reshape the cellular epigenome during development, including methyl group donors (e.g., folate, choline) and bioactive compounds (e.g., polyphenols) is of great importance; however, there is insufficient knowledge of a particular effect of each type of modulator and/or their combination on fetal life. To enhance the quality and safety of food products for proper fetal health and disease prevention in later life, a better understanding of the underlying mechanisms of dietary epigenetic modulators during the critical prenatal period is necessary. This review focuses on the influence of maternal dietary components on DNA methylation, histone modification, and microRNAs, and summarizes current knowledge of the effect and importance of dietary components on epigenetic mechanisms that control the proper expression of genetic information. Evidence reveals that some components in the maternal diet can directly or indirectly affect epigenetic mechanisms. Understanding the underlying mechanisms of how early-life nutritional environment affects the epigenome during development is of great importance for the successful prevention of adult chronic diseases through optimal maternal nutrition.

## 1. Introduction

Accumulating evidence demonstrates clearly that heritable changes in gene expression driven by epigenetic mechanisms play an important role not only in early development, but also in the predisposition to future disease development. Currently, the critical role of epigenetic abnormalities, especially DNA methylation, in the pathogenesis of major human non communicable diseases, including cancer, metabolic syndrome, and cardiovascular and autoimmune disease, is well established [1,2,3,4,5,6]. DNA methylation is indispensable for proper embryonic development as emphasized by Gaudet *et al.* [7]. Early embryonic development is of special interest as it is a crucial period in establishing individual epigenetic marks in the genome [8,9]. Additionally, it has been hypothesized that successful health maintenance and health management in later life relies on better understanding of how early life nutrition affects the epigenome and influences the expression of genetic information and disease etiology [10,11] in adulthood.

**Figure 1:**
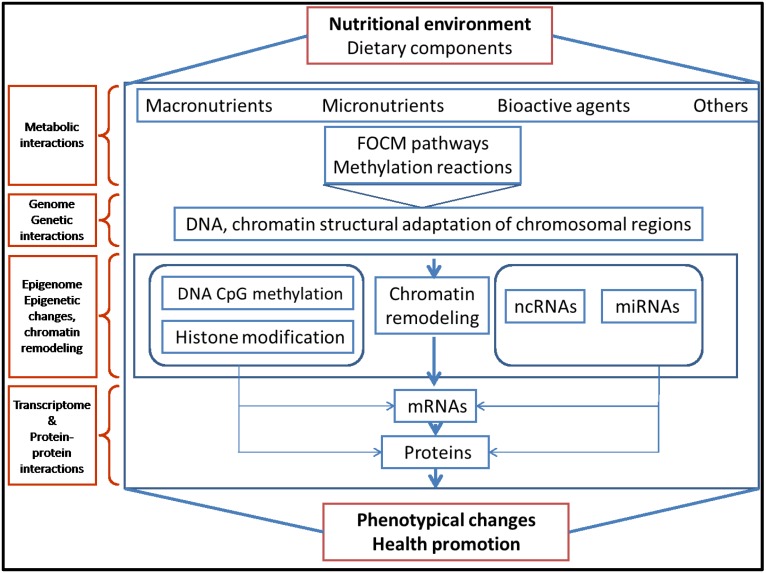
Overview of epigenetic mechanisms providing the link between the nutritional environment and phenotypical changes.

A major finding in the field of nutrition is discovering that dietary components may reshape the genome *in utero* and that epigenetic changes induced during early life may permanently alter the phenotype in the adult organism (Figure 1) [12,13]. A number of reviews have focused on maternal nutrition and its impact on epigenetic mechanisms along with studies addressing different types of exposure, such as nutritional factors, glucocorticoids, and endocrine-disrupting chemicals [11,14,15]. Because a maternal diet and/or early nutrition of the newborn may affect the phenotype later in adulthood [14,15,16], susceptibility of epigenetic mechanisms to the nutritional environment is a critical element in fetal development. However, insufficient knowledge exists in addressing how nutritional factors influence epigenetic mechanisms during fetal development and how to prevent potential negative effects on health. The impact of nutrition on genomic DNA methylation through one-carbon metabolism is well-documented. Specifically, it has been demonstrated that dietary deficiency or excess of the methyl group donors needed for the cellular methylation reactions can alter epigenetic patterns, which may persist for a long period and alter gene expression causing phenotypic changes. In contrast, the impact of diet on other epigenetic mechanisms, including histone modification, chromatin modifying proteins, and microRNA (miRNA) expression is poorly defined. Some dietary components may induce favorable epigenetic effects on the organism (health promoters), while others may cause rather unfavorable or negative epigenetic health effects. In this respect, it is of great importance to identify both the favorable and unfavorable epigenetic impact of dietary components.

This review highlights the nutritional epigenetic aspects of the contemporary maternal diet and summarizes current knowledge of the effect and importance of dietary components on DNA methylation, histone modifications, and miRNA expression in controlling the proper expression of development-related genes. Understanding the underlying mechanisms of how early-life nutritional environment affects our health can be valuable for successful prevention of adult chronic diseases through optimal maternal nutrition.

Dietary components (amino acids, high-fat or high-glucose diet, vitamins, bioactive factors,) can affect genome function and gene expression *in utero* and during early life, influencing epigenetic mechanisms through folate-mediated one-carbon metabolism or transmethylation pathways to affect DNA methylation, histone, or non-coding miRNAs.

## 2. Epigenetic Mechanisms and Epigenome Stability

Epigenetics is defined as heritable changes in gene expression that are not due to any alteration in the primary DNA sequence. Epigenetics (in contrast to genetics, which represents fixed information, *i.e.*, the primary DNA sequence), signifies the way genetic information is organized, maintained, and read. Epigenetic modifications include the best-known and much studied methylation of DNA, modifications of the histone proteins that bind to DNA, the nucleosome positioning along DNA [17], and small and long non-coding RNAs (ncRNAs), including miRNAs.

In mammalian DNA, cytosines in the CpG dinucleotide context are commonly methylated. DNA methylation is involved in the normal development and maintenance of cellular homeostasis and functions in adult organisms, particularly for X-chromosome inactivation in females, genomic imprinting, silencing of repetitive DNA elements, regulation of chromatin structure, and control of gene expression. DNA methylation is well balanced in normal cells; however, it should be noted that genetic [18], environmental [19], and stochastic factors have distinct effects on DNA methylation patterns at individual genomic regions. In addition, DNA methylation at specific CpG-sites can also vary over time within an individual [20,21]. The DNA methylation reaction is catalyzed by a family of DNA methyltransferases (DNMTs) [22]. DNA methylation is initiated and established by means of the *de novo* DNMTs, DNMT3A and DNMT3B [22]. Approximately 70%–90% of CpG dinucleotides in the mammalian genome are methylated; however, CpG sites are not distributed uniformly across the genome [23,24,25] and are concentrated in short regions (<4 kb) of DNA with a high G + C content and a high frequency of CpG dinucleotides called “CpG islands”. In normal cells, CpG sites located in CpG islands are unmethylated. In contrast, most of the remaining CpG sites of the genome are methylated. Cytosine methylation is a stable modification of the genomic DNA and the pattern of DNA methylation is inherited during DNA replication. This epigenetic process also dynamically changes during the lifespan in certain cells and tissues of an organism and is susceptible to nutritional and other environmental influences.

The second well-studied and more complex epigenetic mechanism that regulates chromatin structure and accessibility and transcriptional activities inside a cell involves modifications of histone proteins. These post-translational modifications of histone proteins play an important part in a wide array of cellular processes, including regulation of gene transcription, DNA repair, cell cycle, and metabolic control [26]. The histone modifications function by either influencing chromatin packaging or by recruiting and/or occluding other protein complexes. Histones (H2A, H2B, H3, and H4) are evolutionary conserved proteins that have a globular carboxy-terminal domain critical to a nucleosome formation and a flexible amino-terminal tail that protrudes from the nucleosome core and contacts adjacent nucleosomes in a higher-order structure. The amino-terminal tails of histones are subject to at least eight types of post-translational modifications, including acetylation, methylation, phosphorylation, ubiquitylation, sumoylation, biotinylation, and ADP ribosylation [26]. Acetylation and methylation of histone lysine residues are the most studied post-translational histone modifications.

Typically, histone acetylation, catalyzed by histone acetyltransferase (HAT) enzymes, is associated with the formation of open chromatin structure and active gene transcription. In contrast, histone lysine deacetylation, catalyzed by several different classes of histone deacetylases (HDACs), is associated with gene silencing [27]. For instance, transcriptional activation is associated with acetylation of residues K9 (lysine 9) and K14 (lysine 14), and methylation of residues K4 (lysine 4), K36 (lysine 36) in histone H3. Gene repression has been linked to H3K9, H3K27, and H4K20 methylation [28]. Deacetylated lysines are positively charged and interact strongly with the negatively charged DNA, which leads to chromatin condensation at gene promoters and transcriptional gene repression by limiting access to the transcription machinery [27]. A number of histone-modifying enzymes have been identified, including histone lysine and arginine methyltransferases, histone lysine demethylases, and HAT and HDAC proteins. The altered expression and/or activity of several histone-modifying enzymes has been linked to disease development [29].

Recently, extensive studies have indicated the existence and importance of another mechanism of epigenetic regulation of gene function mediated by means of miRNAs and other small and long ncRNAs. Currently, miRNAs are recognized as major regulatory gatekeepers of protein-coding genes in the human genome. They are small non-coding RNAs, 16–29 nucleotides-long, that function primarily as negative gene regulators at the post-transcriptional level. Following transcription, primary miRNAs form a stem-loop structure, which is recognized by the RNase III-type enzyme Drosha-creating precursor miRNAs. These precursor miRNAs are transported from the nucleus to the cytoplasm by Exportin-5. In the cytoplasm, the pre-miRNAs are further regulated by Dicer, an RNAase III enzyme, generating miRNA:miRNA hybrids. After unwinding, one strand of the duplex is degraded, and another strand is a mature miRNA. miRNAs can induce mRNA cleavage if complementarity to the 3′-untranslated region (3′-UTR) of target mRNAs is perfect, or translational repression if complementarity is imperfect. Currently, more than 2800 mammalian miRNAs that potentially target up to 60% of protein-coding genes involved in cell development and differentiation have been annotated [30,31]. In addition to these miRNAs, 3707 novel miRNAs have been identified recently, many of which are human-specific and tissue specific [31]. This finding indicates that the human genome contains a substantially greater number of uncharacterized miRNAs that may be involved in disease development [31].

All components of the cellular epigenome (*i.e.*, DNA methylation, histone modifications, and miRNAs) are tightly and interdependently connected. For instance, DNA methylation depends on the pattern of histone modifications and functioning of histone modifying proteins. Likewise, the status of histone modifications relies on DNA methylation. Similarly, the expression of many miRNAs is epigenetically regulated either by DNA methylation or histone modifications [32]. On the other hand, several miRNAs directly target DNMTs and other chromatin modifying genes.

## 3. Evidence that Dietary Methyl Group Donors Are Involved in Early Epigenetic Mechanisms

### 3.1. The Folate-Mediated One-Carbon Metabolism and DNA Methylation

**Figure 2:**
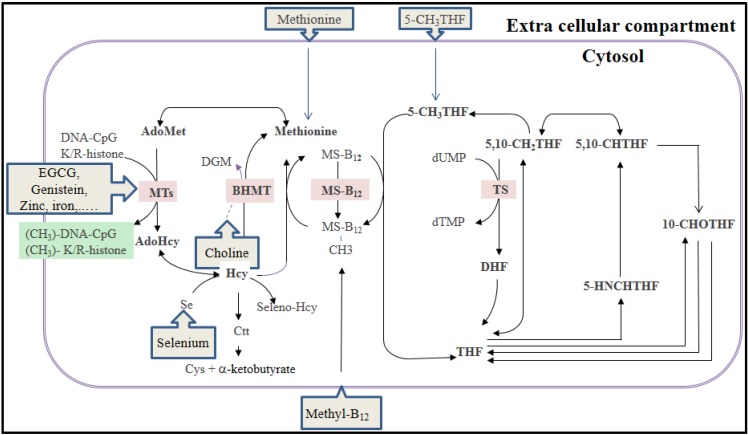
The folate-mediated one-carbon metabolism and interactions with dietary contents. AdoMet: adenosylmethionine, AdoHcy: adenosylhomocysteine, BHMT: Betaine-hydroxymethyl-transferase, DGM: dimethylglycine, EGCG: epigallocatechin-3-gallate, K/R-histone: lysine (K) and arginine (R) in the histone tail, MTs: methyl-transferases; MS-B_12_: methionine synthase linked to the cobalamine (Vitamin B_12_).

Genomic DNA methylation is the addition of a methyl group from the universal methyl donor, *S*-adenosyl-l-methionine (AdoMet) to carbon five in the cytosine pyridine ring, resulting in the formation of 5-methylcytosine (5metC) in DNA (Figure 2). As a consequence, DNA methylation depends upon the availability of methyl groups from AdoMet. This finding has identified the critical role of AdoMet as a key metabolite in the mechanism of DNA methylation. Folate, methionine, choline, betaine, and methylcobalamine affect DNA methylation through the FOCM pathway [33]. The major sources of methyl groups in human foods come from methionine (~10 mmol of methyl/day), 5methylTHF (~5–10 mmol of methyl/day), and from choline (~30 mmoles methyl/day) [34]. Dietary deficiency in any of the factors leads to loss of DNA methylation in humans and experimental animals.

In general, nutrition conditions interfere with the epigenome in, at least, three ways (Table 1). First, nutrients influence the supply of methyl groups for the formation of AdoMet (e.g., methionine supplying or synthesis, homocysteine re-methylation, 5methylTHF supplying, MTHFR enzyme down regulation or activity). Additionally, nutrients modify utilization of methyl groups by mechanisms including shifts in methyl transferase activity in the trans-methylation reaction, and mechanisms related to a DNA demethylation activity. Secondly, nutrients modify chromatin remodeling, or lysine or arginine residues at the N-terminal of histone tails. Finally, nutrients may alter the expression of miRNAs that regulate the level of key FOCM pathway proteins [35,36].

Alterations in the supply of methyl groups appear to be a common mechanism associated with epigenetic aberrations. Methyl group deficiency resulting from dietary methyl source inadequacies, and/or other life-style factors (e.g., alcohol, tobacco, and stress), can lead to global and/or specific DNA methylation changes. For instance, studies on viable yellow (*Avy/a*) mice have shown an association between epigenetic variation in the gene involved in coat coloring and methyl donor supply. Specifically, Wolff *et al.* [37] reported that feeding pregnant agouti mice methyl-supplemented diets alters epigenetic regulation of the offspring. This finding was confirmed in several independent studies [38,39]. The effect of folate deficiency on the phenotype in agouti mice is, probably, the major evidence demonstrating the strong relationship between nutritional factors and epigenetic alterations in the induction of phenotypic generational and trans-generational effects [40,41].

**Table 1:** Epigenetic processes and possible effects of dietary components.

Epigenetic Processes	Molecular Interference
DNA methylation/demethylation	Methyl group supply and availability and alteration of AdoMet synthesis
	Expression of FOCM pathway genes
	Aberrant functioning of methyl-CpG-binding proteins
	DNA-cytosine demethylation
Chromatin remodeling, histones post-translational modifications	Histone (lysine/arginine) methylation or demethylation
	Histone acetylation or deacetylation
	Chromatin remodeling proteins-complex activities
	Others and unknown chromatin modifications
miRNA regulation: activation/inhibition	Specific miRNA activation
	Specific miRNA inhibition

The effect of diet on the phenotype determinism through epigenetic mechanisms has been reported in several independent studies. Specifically, Elango *et al.* [42] and Hunt *et al.* [43] demonstrated that either queen or worker phenotype in honeybees is determined through epigenetic changes in DNA methylation patterns induced by different types of honey. This indicates that the genome uses DNA methylation to control the expression of genes involved in core biological mechanisms. Growing evidence showed the significance of DNA methylation aberrations in the pathogenesis of a number of pathologies. Ghoshal *et al.* [44] reported substantial alterations in the expression of DNMTs and methyl CpG binding proteins at early stages of hepatocarcinogenesis induced by folate and methyl deficiency in Fisher 344 rats. Padmanabhan *et al.* [45] showed the importance of methionine synthase reductase (MSR), an enzyme involved in the utilization of methyl groups from the folate cycle, in intrauterine growth restriction, developmental delay, neural tube, heart, and placental defects, through trans-generational epigenetic inheritance in mice.

Some dietary components (e.g., EGCG, genistein, zinc, iron) may interact with FOCM at the MTs pathways. Choline is a methyl source that contributes to methionine synthesis throughout the BHMT pathway. The methyl groups from 5methylTHF or methylcobalamine (Vitamin B_12_) are provided throughout the methionine synthesis (MS) pathway.

### 3.2. Dietary Methyl Group Donors and Histone Modifications

Several reports have focused on the direct effect of dietary methyl group inadequacy on the modification of histone proteins. Pogribny *et al.* [46] demonstrated that feeding rats a methyl donor-deficient diet resulted in loss of histone H3K9 and H4K20 methylation accompanied by a decreased level of Suv4-20h2 and RIZ1 histone methyltransferases. In contrast, feeding mice a methyl donor-balanced diet up-regulates RIZ1, resulting in greater histone H3K9 methylation [47]. Additionally, Luka *et al.* [48] showed that nuclear lysine-specific demethylase 1 (LSD1), a flavin-containing enzyme that removes the methyl groups from lysines 4 and 9 of histone 3 with the generation of formaldehyde from the methyl group, is a folate-binding protein. Using a natural pentaglutamate form of THF, the authors observed binding with the highest affinity (*K*_d_ = 2.8 μM) to LSD1. The fact that folate participates in the enzymatic demethylation of histones suggests a new role of folate in the epigenetic control of gene expression at histone level.

### 3.3. Dietary Methyl Group Donors and miRNAs

Stone *et al.* [36] applied computational miRNA target prediction methods and Monte Carlo-based statistical analyses to investigate the role of miRNAs in the regulation of folate-mediated one-carbon metabolism pathway and identified two candidates, “master regulators” miRNAs, miR-22 and miR-125, and “master co-regulators” miRNAs, miR-344-5p/484 and miR-488, that may influence the expression of a significant number of genes involved in FOCM. Interestingly, miR-22 and miR-125 were significantly up-regulated in cells grown under low-folate conditions. Although only a few experimental studies showing a link between miRNAs and FOCM, these *in silico* simulations suggest that miRNAs could play an important role in the regulation of the FOCM. Additionally, a recent report by Franchina *et al.* [49] revealed the involvement of mirR-22, miR-24 and miR-34a in folate pathway. Although only few studies showing a link between miRNAs and FOCM, these *in silico* simulations suggest that miRNAs could play an important role in this metabolism regulation. Indeed, it has been demonstrated recently that miR-22 and miR-29b directly target rat *Mthfr* and *Mat1* genes, respectively [50].

### 3.4. Dietary Methyl Group Donors and Fetal Programming

Increased folate demand during pregnancy is necessary to accommodate both fetal development and placental functionality [51,52]. Exogenous stressors in the maternal environment combined with a maladaptation of the placental response result in a small placenta, as is typical of intrauterine growth restriction and preeclampsia [53]. Fryer *et al.* [54] found that levels of folate-associated intermediates in cord blood during late pregnancy are negatively correlated with the level of methylation of LINE-1 repetitive elements in cord lymphocyte samples in offspring of mothers taking daily folic acid supplements during pregnancy. These data support the fact that folate and other one-carbon intermediates may determine clinical programming effects via DNA methylation. Dietary restriction of methyl donors during the periconceptual period results in adverse phenotypes in progeny ewes, which has been associated with changes in the DNA methylation status of ~4% of CpG islands studied in the progeny. These findings imply that modification of the epigenetic status of a small subset of genes may be a cause of the programming events [55].

Fetal life is characterized by a tremendous plasticity and ability to respond to environmental and lifestyle factors, including maternal nutrition. From the single cell to the blastocyst stage of the embryogenesis many changes in global DNA methylation and histone modifications occur. DNA methylation is reduced progressively with cleavage divisions; DNMT1 protein is seemingly excluded from the nucleus during the first three cleavage divisions [56] accounting for the loss of DNA methylation by a passive mechanism. Almost all DNA methylation patterns are erased as preimplantational embryo development proceeds [56], and many different types of sequences lose methylation at this stage. DNMT3 predominantly mediates *de novo* DNA methylation, in which the hypermethylated inner cell mass will give rise to the embryo while trophoblastic cells remain hypomethylated. Thus, embryonic development is characterized by a wave of demethylation followed by re-establishment of methylation patterns in developing embryos, which can induce the removal of acquired epigenetic modification. Exposure to a high-fat diet *in utero* might cause a metabolic syndrome-like phenomenon through epigenetic modifications of the expression of insulin-like growth factor 2 (*IGF2*), a candidate in developmental programming and determinant of later adult disease risk [12,57].

Taking into account these observations, an altered nutritional status during early life could produce changes in epigenetic marks that have lifelong phenotypic consequences [39,58]. Identifying critical windows of sensitivity to epigenetic modulation by dietary compounds is needed to prevent the development of metabolic diseases.

## 4. Epigenetic Effects of Common Macronutrients Derivatives, Micronutrients and Bioactive Compounds in Modern Diet

Some of the most investigated dietary factors capable of interfering with the epigenetic processes have been reported [59]. The effects of macronutrient derivatives (e.g., methionine, choline, and betaine) [60,61,62,63], and micronutrients such as vitamins (e.g., B-vitamins, D vitamin, and retinoic acid [64,65,66,67], microminerals or trace elements (e.g., iron, zinc and selenium) [68,69,70] and bioactive compounds (e.g., phytochemicals including polyphenols) [71,72] on epigenetic processes are summarized in Table 2, Table 3 and Table 4, respectively. These dietary compounds are an integral part of everyday nutrition in human populations worldwide, are frequently present in maternal diets, and have been reported to influence epigenetic mechanisms (Figure 2). For example, an inadequate dietary protein amount, methionine-deficient diet, high-fat diet, or high-glucose diet have been reported to modulate the epigenetic process (Table 2). The phytochemicals lycopene in tomatoes, genistein in soybeans, resveratrol in grapes and berries, sulforaphane in broccoli, epigallocatechin-3-gallate (EGCG) in green tea, or curcumin and allyl sulfur compounds present in spices are among a growing list of agents used in the modern diet that might affect epigenetic mechanisms. We have summarized bioactive agents that are most frequently present in the maternal diet (Table 4). For instance, green tea commonly used by mothers in European countries contains high amount of EGCG. Those who consume black tea or coffee more regularly, have a greater intake of the respective major components of these beverages, theophylline or caffeic acid. Coffee polyphenols such as caffeic acid or chlorogenic acid are catechol-containing polyphenols that act in a similar way to the tea polyphenols. However, the specific epigenetic modulations of these phenolic compounds are not well documented. They can be demethylating agents, inhibiting DNMT1-catalyzed DNA methylation in a concentration-dependent manner, predominantly through a non-competitive mechanism [73].

Several other dietary epigenetic modulators have been investigated for further understanding of molecular mechanisms underpinning epigenetic effects in the context of chemoprevention [74,75,76,77,78]. Some have shown potential to reverse methylation-induced silencing and change the expression of various genes (*i.e.*, DNMT inhibitors). For example, EGCG of green tea extract is a demethylating agent that inhibits catechol-*O*-methyltransferase (COMT), the enzyme responsible for the inactivation of catechol molecules [79]. This enzyme introduces the methyl group from AdoMet onto the catecholamine group forming AdoHcy, a potent inhibitor of DNMTs. On the other hand, EGCG can form hydrogen bonds with different residues in the catalytic pocket of DNMTs acting as a direct inhibitor of DNMT1. Lycopene, a bright red carotene and carotenoid pigment found in tomatoes and other red fruits and vegetables modulates the expression of numerous genes relevant to cell cycle control [80]; however, lycopene and apo-10'-lycopenal are not effective demethylating agents of *GSTP1* in the human LNCaP prostate cancer cell line [81]. It has been demonstrated that treatment of the MV4-11 leukemia cell line with curcumin decreases global DNA methylation [82]. Genistein, a major phytoestrogen in soybeans, induces a dose-dependent inhibition of DNMTs. Furthermore, prenatal exposure to genistein affects fetal erythropoiesis and exerts lifelong alterations in gene expression and DNA methylation of hematopoietic cells [83]. Sulforaphane, a bioactive component of cruciferous vegetables, down-regulates DNMT1 and induces demethylation of the Cyclin D2 (*CCND2*) gene in the human colorectal adenocarcinoma Caco-2 cell line [84]. A wide variety of cruciferous vegetables contain isothiocyanate compounds, which are known to affect the epigenome. For example, isothiocyanates, metabolites of glucosinolates present in cruciferous vegetables, lead to demethylation and re-expression of *GSTP1* [85]. Resveratrol, a polyphenol phytoalexin in grape peel and a weak inhibitor of DNMT activity, increases the ability of adenosine analogues to reduce DNA methylation and increase the expression of *RARβ2* in human breast cancer MCF-7 cells [86].

A number of bioactive agents, in addition to their ability to affect DNA methylation, induce changes in histone modifications and regulate gene expression [87]. For instance, EGCG has been shown to affect HAT and HDAC activities as it has been reported to be the most potent HAT inhibitor [88]. Quercetin, a flavonoid abundant in onions, green tea, apples, and berries, has been reported to exhibit potential to activate HATs and inhibit HDACs [89]. Resveratrol, butyrate, sulforaphane, and diallyl sulfide inhibit HDACs, whereas curcumin inhibits HAT activity via covalent binding to HAT enzymes. In addition to the HAT-inhibitory effect, it has been shown that curcumin also prevents histone hyperacetylation by induction of HDACs [73]. Although it is accepted that curcumin functions as a histone modifier, its activity toward other histone modifying enzymes such as HDACs, SIRTs, and HMTs remains controversial.

In regard to miRNAs, the understanding of the effect of dietary components on miRNA expression is currently limited; however, few studies have identified effects on specific miRNA targets. Ross and Davis [90] reported that bioactive food components protect against cancer through modulation of miRNA expression. Natural food compounds, including EGCG, curcumin, genistein, sulforaphane, and resveratrol, have anticancer properties through miRNA regulation [91].

## 5. Future Considerations of Dietary Epigenetic Modulators

It is well-established that maternal diet may have a lifelong effect on an offspring’s genome and potentially influence susceptibility to complex diseases in adulthood. In light of this, awareness of nutrient supply during pregnancy is currently unsatisfactory and should be improved to protect against adverse fetal programming and susceptibility to complex diseases in adulthood. Emerging evidence has revealed that some components in the maternal diet can directly or indirectly affect DNA methylation, histone modifications, or miRNA expression (Figure 1). The field of nutritional epigenetics is growing rapidly and we are only beginning to understand the complexity of gene regulation through this mechanism. However, it is clear that some dietary components contribute actively to epigenetic mechanisms and modulate the expression of a number of genes. The particular effect of each type of those dietary epigenetic modulators or their combination on fetal life remains questionable because of insufficient data and serious methodological limitations. Additionally, it is conceivable to anticipate that new or unsuspected biological activity may emerge from a manufactured or engineered diet. A major feature of epigenetic mechanisms is their modulation responsiveness, which makes possible early-life nutritional intervention to modify long-term disease risk. However, this modification may be possible only for specific windows of epigenetic change, which makes the identification of such windows of great importance. Additionally, improving the environment to which the fetus/infant is exposed during critical windows of development may be as important as other public health efforts to prevent long term diseases.

Although it is conceivable that dietary components should positively affect maternal health and fetal development, the first question arising at this level is of the influence on fetal programming in an excess or deficiency of dietary factors. The second question is on the impact of these “dietary epigenetic modulators” on an individual genetically predisposed (individual susceptibility) for developing a particular disease, including transgenerational effects. Finally, the question of the combined effects of different dietary epigenetic modulators or chronic exposures to a dietary epigenetic modulator requires investigation, taking into account genetic profiles characterized by the presence of identified functional single nucleotide polymorphisms [92,93].

**Table 2:** Relevant studies on macronutrients, macronutrient derivatives (choline, betaine), nutrition condition and epigenetic interference.

Macronutrient	Dietary Compounds	Epigenetic Interference		
	Nutrition Condition	DNMT Pathway	Histone Modification	microRNAs
Protein	Low protein diet	[93,94]	[94]	[95]
Lipids	High-fat	[96,97,98,99]	[12,100]	[101,102]
	Fatty acids	[103]		
	Choline	[104,105]	[106]	[107]
	Betaine	[108]		
Carbohydrate	High-glucose	[109]	[110,111]	
	Fiber: butyrate	[112,113,114]	[112]	
Nutrition conditions	Undernutrition: Calorie restriction	[113,115]	[113,114]	
	Overfeeding: High calories	[116]		

**Table 3:** The effects of micronutrients, vitamins, and trace elements on epigenetic processes.

Micronutrient	Dietary Compound *Source*	Epigenetic Interference		
		DNMT, DNA Methylation	Histone Modification	miRNAs
Vitamins	Folate/methyl-deficient diet *Vegetables, cereals, yeast*	[117,118,119,120]	[121,122,123]	[124,125]
	Ascorbate *Fruits and vegetables*	[126]		
	Retinoic acid *Yellow and orange fruits*	[65,127,128]	[129,130,131,132]	[131,133]
	Biotin *Yeast, egg yolk, grains*		[122,134]	
	Tocopherol (vitamine E) *Grains, nuts and oils*	[135]	[136]	
Trace elements	Zinc *Meat, seafood, whole grains*	[137]		
	Copper *Seafood, nuts, legumes, meats*			[138]
	Selenium *Meat, seafood, whole grains*	[139,140]		

**Table 4:** The effects of dietary phytochemicals effect on epigenetic processes summarized from literature *.

Dietary Compounds *Source*	Epigenetic Interference		
	DNMT Pathway	Histone Modification	miRNA
Epigallocatechin-3-gallate *Green tea*	Known		
6-methoxy-2^E^,9^E^-humuladien-8-one *Ginger*		Known	
Allylmercaptant, allyl-derivates *Garlic*	Known	Known	
Anacardic acid *Cashew nut*	Known		
Biochanin A *Soy*	Known	Known	
Caffeic acid, chlorogenic acid *Coffee*	Known	Known	
Catechin *Green tea*	Known	Known	
Coumaric acid, cinnamic acid *Cinnamon*	Known	Known	
Curcumin *Curcuma*	Known	Known	Known
Epicatechin *Apples, cocoa*	Known		
Epigallocatechin-3-gallate *Green tea*	Known	Known	
Genistein *Soy*	Known	Known	Known
Isothiocyanates *Broccoli*	Known		
Lycopene *Tomatoes, apricots*	Known	Known	
Protocatechuric acid *Olives*	Known		
QuercetinC *itrus, buckwheat, apple, berries, tea*	Known	Known	
Resveratrol *Grape, blueberries, peanut*	Known	Known	
Rosmarinic *Rosemary*	Known		
Sinapic acid *Mustard*	Known		
Sulforaphane *Broccoli*	Known	Known	
Syringic acid *Red grape*	Known	Known	
Theophylline *Black and green tea*		Known	

*: Relevant references: [71,83,85,87,89,141,142,143,144].

Depending on the tissues, hyper- or hypo-methylation, or other epigenetic modifications induced by diet may be beneficial for some genes, but deleterious for the normal expression of other genes. In this context, a strategy for food safety and disease prevention should be considered and exercised in interpreting and/or extrapolating the results of experimental studies and *in vitro* studies of “dietary epigenetic modulators” to humans. Future research will help to enhance our understanding of their impacts on the nutritional programming of epigenetic states in early life and effect on biological function. One anticipated result is that these “dietary epigenetic modulators” could be identified as nutraceuticals and the next, and most important step, would be to determine the effective and optimal doses to achieve various beneficial effects during the mother-child metabolic interaction. The daily diet of humans consists of approximately 50–60 mmol of methyl groups. Perhaps, we need to reconsider the optimal need during the perinatal period and investigate events that can influence epigenome, inducing significant long-term health effects in adulthood. Determining the impact of domestic cooking modes or industrial treatment on the functional quality of these “dietary epigenetic modulators” also requires investigation. Addressing these questions may open up a new research field of nutritional epigenetics that should contribute to public policy guidance that will teach optimal, rather than minimal, dose levels to meet both fetal and maternal needs and health.

## 6. Conclusions

In conclusion, dietary components have a strong impact upon epigenetic processes and metabolic programing during sensitive periods of fetal and early postnatal periods. Understanding how nutritional manipulations alter the epigenetic machinery to affect metabolic genes may help to better identify strategies for the successful prevention of adult chronic diseases.

## References

[1] Baylin S.B., Jones P.A. (2011). A decade of exploring the cancer epigenome-biological and translational implications. Nat. Rev. Cancer.

[2] Rodenhiser D., Mann M. (2006). Epigenetics and human disease: Translating basic biology into clinical applications. CMAJ.

[3] Hewagama A., Richardson B. (2009). The genetics and epigenetics of autoimmune diseases. J. Autoimmun..

[4] Lopez-Pedrera C., Perez-Sanchez C., Ramos-Casals M., Santos-Gonzalez M., Rodriguez-Ariza A., Cuadrado M.J. (2012). Cardiovascular risk in systemic autoimmune diseases: Epigenetic mechanisms of immune regulatory functions. Clin. Dev. Immunol..

[5] Barres R., Zierath J.R. (2011). DNA methylation in metabolic disorders. Am. J. Clin. Nutr..

[6] Brookes E., Shi Y. (2014). Diverse epigenetic mechanisms of human disease. Annu. Rev. Genet..

[7] Gaudet F., Hodgson J.G., Eden A., Jackson-Grusby L., Dausman J., Gray J.W., Leonhardt H., Jaenisch R. (2003). Induction of tumors in mice by genomic hypomethylation. Science.

[8] Reik W., Dean W., Walter J. (2001). Epigenetic reprogramming in mammalian development. Science.

[9] Smith Z.D., Meissner A. (2013). DNA methylation: Roles in mammalian development. Nat. Rev. Genet..

[10] Saffery R., Novakovic B. (2014). Epigenetics as the mediator of fetal programming of adult onset disease: What is the evidence?. Acta Obstet. Gynecol. Scand..

[11] Jiang X., West A.A., Caudill M.A. (2014). Maternal choline supplementation: A nutritional approach for improving offspring health?. Trends Endocrinol. Metab..

[12] Masuyama H., Hiramatsu Y. (2012). Effects of a high-fat diet exposure in utero on the metabolic syndrome-like phenomenon in mouse offspring through epigenetic changes in adipocytokine gene expression. Endocrinology.

[13] Thornburg K.L., Shannon J., Thuillier P., Turker M.S. (2010). *In utero* life and epigenetic predisposition for disease. Adv. Genet..

[14] Hogg K., Price E.M., Hanna C.W., Robinson W.P. (2012). Prenatal and perinatal environmental influences on the human fetal and placental epigenome. Clin. Pharmacol. Ther..

[15] Mathers J.C. (2007). Early nutrition: Impact on epigenetics. Forum Nutr..

[16] Szyf M. (2009). The early life environment and the epigenome. Biochim. Biophys. Acta.

[17] Sharma S., Kelly T.K., Jones P.A. (2010). Epigenetics in cancer. Carcinogenesis.

[18] Bell J.T., Pai A.A., Pickrell J.K., Gaffney D.J., Pique-Regi R., Degner J.F., Gilad Y., Pritchard J.K. (2011). DNA methylation patterns associated with genetic and gene expression variation in HapMap cell lines. Genome Biol..

[19] Pogribny I.P., Rusyn I. (2013). Environmental toxicants, epigenetics, and cancer. Adv. Exp. Med. Biol..

[20] Fraga M.F., Ballestar E., Paz M.F., Ropero S., Setien F., Ballestar M.L., Heine-Suñer D., Cigudosa J.C., Urioste M., Benitez J. (2005). Epigenetic differences arise during the lifetime of monozygotic twins. Proc. Natl. Acad. Sci. USA.

[21] Talens R.P., Boomsma D.I., Tobi E.W., Kremer D., Jukema J.W., Willemsen G., Putter H., Slagboom P.E., Heijmans B.T. (2010). Variation, patterns, and temporal stability of DNA methylation: Considerations for epigenetic epidemiology. FASEB J..

[22] Ooi S.K., O’Donnell A.H., Bestor T.H. (2009). Mammalian cytosine methylation at a glance. J. Cell Sci..

[23] Das R., Dimitrova N., Xuan Z., Rollins R.A., Haghighi F., Edwards J.R., Ju J., Bestor T.H., Zhang M.Q. (2006). Computational prediction of methylation status in human genomic sequences. Proc. Natl. Acad. Sci. USA.

[24] Rollins R.A., Haghighi F., Edwards J.R., Das R., Zhang M.Q., Ju J., Bestor T.H. (2006). Large-scale structure of genomic methylation patterns. Genome Res..

[25] Mikkelsen T.S., Ku M., Jaffe D.B., Issac B., Lieberman E., Giannoukos G., Alvarez P., Brockman W., Kim T.K., Koche R.P. (2007). Genome-wide maps of chromatin state in pluripotent and lineage-committed cells. Nature.

[26] Kouzarides T. (2007). Chromatin modifications and their function. Cell.

[27] Ropero S., Esteller M. (2007). The role of histone deacetylases (HDACs) in human cancer. Mol. Oncol..

[28] Iizuka M., Smith M.M. (2003). Functional consequences of histone modifications. Curr. Opin. Genet. Dev..

[29] Butler J.S., Koutelou E., Schibler A.C., Dent S.Y. (2012). Histone-modifying enzymes: Regulators of developmental decisions and drivers of human disease. Epigenomics.

[30] Friedman R.C., Farh K.K., Burge C.B., Bartel D.P. (2009). Most mammalian mRNAs are conserved targets of microRNAs. Genome Res..

[31] Londin E., Loher P., Telonis A.G., Quann K., Clark P., Jing Y., Hatzimichael E., Kirino Y., Honda S., Lally M. (2015). Analysis of 13 cell types reveals evidence for the expression of numerous novel primate- and tissue-specific microRNAs. Proc. Natl. Acad. Sci. USA.

[32] Denis H., Ndlovu M.N., Fuks F. (2011). Regulation of mammalian DNA methyltransferases: A route to new mechanisms. EMBO Rep..

[33] Waterland R.A. (2006). Assessing the effects of high methionine intake on DNA methylation. J. Nutr..

[34] Niculescu M.D., Zeisel S.H. (2002). Diet, methyl donors and DNA methylation: Interactions between dietary folate, methionine and choline. J. Nutr..

[35] Hardy T.M., Tollefsbol T.O. (2011). Epigenetic diet: Impact on the epigenome and cancer. Epigenomics.

[36] Stone N., Pangilinan F., Molloy A.M., Shane B., Scott J.M., Ueland P.M., Mills J.L., Kirke P.N., Sethupathy P., Brody L.C. (2011). Bioinformatic and genetic association analysis of microrna target sites in one-carbon metabolism genes. PLoS ONE.

[37] Wolff G.L., Kodell R.L., Moore S.R., Cooney C.A. (1998). Maternal epigenetics and methyl supplements affect agouti gene expression in *Avy/a* mice. FASEB J..

[38] Cooney C.A., Dave A.A., Wolff G.L. (2002). Maternal methyl supplements in mice affect epigenetic variation and DNA methylation of offspring. J. Nutr..

[39] Waterland R.A., Jirtle R.L. (2003). Transposable elements: Targets for early nutritional effects on epigenetic gene regulation. Mol Cell Biol.

[40] Waterland R.A., Travisano M., Tahiliani K.G., Rached M.T., Mirza S. (2008). Methyl donor supplementation prevents transgenerational amplification of obesity. Int. J. Obes. (Lond.).

[41] Cropley J.E., Suter C.M., Beckman K.B., Martin D.I. (2006). Germ-line epigenetic modification of the murine A*^vy^* allele by nutritional supplementation. Proc. Natl. Acad. Sci. USA.

[42] Elango N., Hunt B.G., Goodisman M.A., Yi S.V. (2009). DNA methylation is widespread and associated with differential gene expression in castes of the honeybee, *Apis mellifera*. Proc. Natl. Acad. Sci. USA.

[43] Hunt B.G., Brisson J.A., Yi S.V., Goodisman M.A. (2010). Functional conservation of DNA methylation in the pea aphid and the honeybee. Genome Biol. Evol..

[44] Ghoshal K., Li X., Datta J., Bai S., Pogribny I., Pogribny M., Huang Y., Young D., Jacob S.T. (2006). A folate- and methyl-deficient diet alters the expression of DNA methyltransferases and methyl CpG binding proteins involved in epigenetic gene silencing in livers of F344 rats. J. Nutr..

[45] Padmanabhan N., Jia D., Geary-Joo C., Wu X., Ferguson-Smith A.C., Fung E., Bieda M.C., Snyder F.F., Gravel R.A., Cross J.C. (2013). Mutation in folate metabolism causes epigenetic instability and transgenerational effects on development. Cell.

[46] Pogribny I.P., Tryndyak V.P., Muskhelishvili L., Rusyn I., Ross S.A. (2007). Methyl deficiency, alterations in global histone modifications, and carcinogenesis. J. Nutr..

[47] Zhou W., Alonso S., Takai D., Lu S.C., Yamamoto F., Perucho M., Huang S. (2008). Requirement of RIZ1 for cancer prevention by methyl-balanced diet. PLoS ONE.

[48] Luka Z., Moss F., Loukachevitch L.V., Bornhop D.J., Wagner C. (2011). Histone demethylase LSD1 is a folate-binding protein. Biochemistry.

[49] Franchina T., Amodeo V., Bronte G., Savio G., Ricciardi G.R., Picciotto M., Russo A., Giordano A., Adamo V. (2014). Circulating miR-22, miR-24 and miR-34a as novel predictive biomarkers to pemetrexed-based chemotherapy in advanced non-small cell lung cancer. J. Cell Physiol..

[50] Koturbash I., Melnyk S., James S.J., Beland F.A., Pogribny I.P. (2013). Role of epigenetic and miR-22 and miR-29b alterations in the downregulation of *Mat1a* and *Mthfr* genes in early preneoplastic livers in rats induced by 2-acetylaminofluorene. Mol. Carcinog..

[51] Novakovic B., Saffery R. (2012). The ever growing complexity of placental epigenetics—Role in adverse pregnancy outcomes and fetal programming. Placenta.

[52] Yajnik C.S., Deshmukh U.S. (2012). Fetal programming: Maternal nutrition and role of one-carbon metabolism. Rev. Endocr. Metab. Disord..

[53] Longtine M.S., Nelson D.M. (2011). Placental dysfunction and fetal programming: The importance of placental size, shape, histopathology, and molecular composition. Semin. Reprod. Med..

[54] Fryer A.A., Nafee T.M., Ismail K.M., Carroll W.D., Emes R.D., Farrell W.E. (2009). LINE-1 DNA methylation is inversely correlated with cord plasma homocysteine in man: A preliminary study. Epigenetics.

[55] Sinclair K.D., Lea R.G., Rees W.D., Young L.E. (2007). The developmental origins of health and disease: Current theories and epigenetic mechanisms. Soc. Reprod. Fertil. Suppl..

[56] Probst A.V., Dunleavy E., Almouzni G. (2009). Epigenetic inheritance during the cell cycle. Nat. Rev. Mol. Cell Biol..

[57] Jirtle R.L., Skinner M.K. (2007). Environmental epigenomics and disease susceptibility. Nat. Rev. Genet..

[58] Sinclair K.D., Singh R. (2007). Modelling the developmental origins of health and disease in the early embryo. Theriogenology.

[59] Shenderov B.A., Midtvedt T. (2014). Epigenomic programing: A future way to health?. Microb. Ecol. Health Dis..

[60] Wilkinson J. (2012). High maternal choline intake may prevent the development of stress-related disorders through epigenetic mechanisms. Epigenomics.

[61] Jiang X., Yan J., West A.A., Perry C.A., Malysheva O.V., Devapatla S., Pressman E., Vermeylen F., Caudill M.A. (2012). Maternal choline intake alters the epigenetic state of fetal cortisol-regulating genes in humans. FASEB J..

[62] Zeisel S.H. (2012). Dietary choline deficiency causes DNA strand breaks and alters epigenetic marks on DNA and histones. Mutat. Res..

[63] Oliva J., Bardag-Gorce F., Li J., French B.A., Nguyen S.K., Lu S.C., French S.W. (2009). Betaine prevents Mallory-Denk body formation in drug-primed mice by epigenetic mechanisms. Exp. Mol. Pathol..

[64] Wei L.N. (2013). Non-canonical activity of retinoic acid in epigenetic control of embryonic stem cell. Transcription.

[65] Cheong H.S., Lee H.C., Park B.L., Kim H., Jang M.J., Han Y.M., Kim S.Y., Kim Y.S., Shin H.D. (2010). Epigenetic modification of retinoic acid-treated human embryonic stem cells. BMB Rep..

[66] Loewy A.D., Niles K.M., Anastasio N., Watkins D., Lavoie J., Lerner-Ellis J.P., Pastinen T., Trasler J.M., Rosenblatt D.S. (2009). Epigenetic modification of the gene for the vitamin B(12) chaperone MMACHC can result in increased tumorigenicity and methionine dependence. Mol. Genet. Metab..

[67] Karlic H., Varga F. (2011). Impact of vitamin D metabolism on clinical epigenetics. Clin. Epigenet..

[68] Tian X., Diaz F.J. (2013). Acute dietary zinc deficiency before conception compromises oocyte epigenetic programming and disrupts embryonic development. Dev. Biol..

[69] Kurita H., Ohsako S., Hashimoto S., Yoshinaga J., Tohyama C. (2013). Prenatal zinc deficiency-dependent epigenetic alterations of mouse metallothionein-2 gene. J. Nutr. Biochem..

[70] Hu Y., McIntosh G.H., Le Leu R.K., Nyskohus L.S., Woodman R.J., Young G.P. (2013). Combination of selenium and green tea improves the efficacy of chemoprevention in a rat colorectal cancer model by modulating genetic and epigenetic biomarkers. PLoS ONE.

[71] Shankar S., Kumar D., Srivastava R.K. (2013). Epigenetic modifications by dietary phytochemicals: Implications for personalized nutrition. Pharmacol. Ther..

[72] Parasramka M.A., Ho E., Williams D.E., Dashwood R.H. (2012). MicroRNAs, diet, and cancer: New mechanistic insights on the epigenetic actions of phytochemicals. Mol. Carcinog..

[73] Lee W.J., Zhu B.T. (2006). Inhibition of DNA methylation by caffeic acid and chlorogenic acid, two common catechol-containing coffee polyphenols. Carcinogenesis.

[74] Lambert J.D., Yang C.S. (2003). Cancer chemopreventive activity and bioavailability of tea and tea polyphenols. Mutat. Res..

[75] Wolfram S., Raederstorff D., Preller M., Wang Y., Teixeira S.R., Riegger C., Weber P. (2006). Epigallocatechin gallate supplementation alleviates diabetes in rodents. J. Nutr..

[76] Wolfram S., Wang Y., Thielecke F. (2006). Anti-obesity effects of green tea: From bedside to bench. Mol. Nutr. Food Res..

[77] Kopelovich L., Crowell J.A., Fay J.R. (2003). The epigenome as a target for cancer chemoprevention. J. Natl. Cancer Inst..

[78] Huang J., Plass C., Gerhauser C. (2011). Cancer chemoprevention by targeting the epigenome. Curr. Drug Targets.

[79] Lu H., Meng X., Yang C.S. (2003). Enzymology of methylation of tea catechins and inhibition of catechol-*O*-methyltransferase by (−)-epigallocatechin gallate. Drug Metab. Dispos..

[80] Gerhauser C. (2013). Cancer chemoprevention and nutriepigenetics: State of the art and future challenges. Top. Curr. Chem..

[81] Liu A.G., Erdman J.W. (2011). Lycopene and apo-10ʹ-lycopenal do not alter DNA methylation of GSTP1 in LNCaP cells. Biochem. Biophys. Res. Commun..

[82] Yu J., Peng Y., Wu L.C., Xie Z., Deng Y., Hughes T., He S., Mo X., Chiu M., Wang Q.E. (2013). Curcumin down-regulates DNA methyltransferase 1 and plays an anti-leukemic role in acute myeloid leukemia. PLoS ONE.

[83] Vanhees K., Coort S., Ruijters E.J., Godschalk R.W., van Schooten F.J., Barjesteh van Waalwijk van Doorn-Khosrovani S. (2011). Epigenetics: Prenatal exposure to genistein leaves a permanent signature on the hematopoietic lineage. FASEB J..

[84] Hsu A., Wong C.P., Yu Z., Williams D.E., Dashwood R.H., Ho E. (2011). Promoter de-methylation of Cyclin D2 by sulforaphane in prostate cancer cells. Clin. Epigenet..

[85] Wang L.G., Beklemisheva A., Liu X.M., Ferrari A.C., Feng J., Chiao J.W. (2007). Dual action on promoter demethylation and chromatin by an isothiocyanate restored GSTP1 silenced in prostate cancer. Mol. Carcinog..

[86] Stefanska B., Rudnicka K., Bednarek A., Fabianowska-Majewska K. (2010). Hypomethylation and induction of retinoic acid receptor beta 2 by concurrent action of adenosine analogues and natural compounds in breast cancer cells. Eur. J. Pharmacol..

[87] Link A., Balaguer F., Goel A. (2010). Cancer chemoprevention by dietary polyphenols: Promising role for epigenetics. Biochem. Pharmacol..

[88] Lee Y.H., Kwak J., Choi H.K., Choi K.C., Kim S., Lee J., Jun W., Park H.J., Yoon H.G. (2012). EGCG suppresses prostate cancer cell growth modulating acetylation of androgen receptor by anti-histone acetyltransferase activity. Int. J. Mol. Med..

[89] Lee W.J., Chen Y.R., Tseng T.H. (2011). Quercetin induces FASL-related apoptosis, in part, through promotion of histone H3 acetylation in human leukemia HL-60 cells. Oncol. Rep..

[90] Ross S.A., Davis C.D. (2014). The emerging role of microRNAs and nutrition in modulating health and disease. Annu. Rev. Nutr..

[91] Wang Y., Li Y., Liu X., Cho W.C. (2013). Genetic and epigenetic studies for determining molecular targets of natural product anticancer agents. Curr. Cancer Drug Targets.

[92] Williams S.R., Yang Q., Chen F., Liu X., Keene K.L., Jacques P., Chen W.M., Weinstein G., Hsu F.C., Beiser A. (2014). Genome-wide meta-analysis of homocysteine and methionine metabolism identifies five one carbon metabolism loci and a novel association of ALDH1L1 with ischemic stroke. PLoS Genet..

[93] Shea T.B., Rogers E. (2014). Lifetime requirement of the methionine cycle for neuronal development and maintenance. Curr. Opin. Psychiatry.

[94] Lillycrop K.A., Slater-Jefferies J.L., Hanson M.A., Godfrey K.M., Jackson A.A., Burdge G.C. (2007). Induction of altered epigenetic regulation of the hepatic glucocorticoid receptor in the offspring of rats fed a protein-restricted diet during pregnancy suggests that reduced DNA methyltransferase-1 expression is involved in impaired DNA methylation and changes in histone modifications. Br. J. Nutr..

[95] Sene Lde B., Mesquita F.F., de Moraes L.N., Santos D.C., Carvalho R., Gontijo J.A., Boer P.A. (2013). Involvement of renal corpuscle microRNA expression on epithelial-to-mesenchymal transition in maternal low protein diet in adult programmed rats. PLoS ONE.

[96] Gillberg L., Jacobsen S.C., Ronn T., Brons C., Vaag A. (2014). PPARGC1A DNA methylation in subcutaneous adipose tissue in low birth weight subjects—Impact of 5 days of high-fat overfeeding. Metabolism.

[97] Langie S.A., Achterfeldt S., Gorniak J.P., Halley-Hogg K.J., Oxley D., van Schooten F.J., Godschalk R.W., McKay J.A., Mathers J.C. (2013). Maternal folate depletion and high-fat feeding from weaning affects DNA methylation and DNA repair in brain of adult offspring. FASEB J..

[98] Jacobsen S.C., Brons C., Bork-Jensen J., Ribel-Madsen R., Yang B., Lara E., Hall E., Calvanese V., Nilsson E., Jorgensen S.W. (2012). Effects of short-term high-fat overfeeding on genome-wide DNA methylation in the skeletal muscle of healthy young men. Diabetologia.

[99] Gallou-Kabani C., Gabory A., Tost J., Karimi M., Mayeur S., Lesage J., Boudadi E., Gross M.S., Taurelle J., Vige A. (2010). Sex- and diet-specific changes of imprinted gene expression and DNA methylation in mouse placenta under a high-fat diet. PLoS ONE.

[100] Aagaard-Tillery K.M., Grove K., Bishop J., Ke X., Fu Q., McKnight R., Lane R.H. (2008). Developmental origins of disease and determinants of chromatin structure: Maternal diet modifies the primate fetal epigenome. J. Mol. Endocrinol..

[101] Cirera S., Birck M., Busk P.K., Fredholm M. (2010). Expression profiles of miRNA-122 and its target cat1 in minipigs (*Sus scrofa*) fed a high-cholesterol diet. Comp. Med..

[102] Takanabe R., Ono K., Abe Y., Takaya T., Horie T., Wada H., Kita T., Satoh N., Shimatsu A., Hasegawa K. (2008). Up-regulated expression of microRNA-143 in association with obesity in adipose tissue of mice fed high-fat diet. Biochem. Biophys. Res. Commun..

[103] Burdge G.C., Lillycrop K.A. (2014). Fatty acids and epigenetics. Curr. Opin. Clin. Nutr. Metab. Care.

[104] Niculescu M.D., Craciunescu C.N., Zeisel S.H. (2006). Dietary choline deficiency alters global and gene-specific DNA methylation in the developing hippocampus of mouse fetal brains. FASEB J..

[105] Medici V., Shibata N.M., Kharbanda K.K., Islam M.S., Keen C.L., Kim K., Tillman B., French S.W., Halsted C.H., LaSalle J.M. (2014). Maternal choline modifies fetal liver copper, gene expression, DNA methylation, and neonatal growth in the tx-j mouse model of Wilson disease. Epigenetics.

[106] Mehedint M.G., Niculescu M.D., Craciunescu C.N., Zeisel S.H. (2010). Choline deficiency alters global histone methylation and epigenetic marking at the Re1 site of the calbindin 1 gene. FASEB J..

[107] Wang B., Majumder S., Nuovo G., Kutay H., Volinia S., Patel T., Schmittgen T.D., Croce C., Ghoshal K., Jacob S.T. (2009). Role of microRNA-155 at early stages of hepatocarcinogenesis induced by choline-deficient and amino acid-defined diet in C57BL/6 mice. Hepatology.

[108] Pellanda H. (2013). Betaine homocysteine methyltransferase (BHMT)-dependent remethylation pathway in human healthy and tumoral liver. Clin. Chem. Lab. Med..

[109] Li M., Cao L., Yang Y. (2014). The role of epigenetic modification in glucose and lipid metabolism. Yi Chuan.

[110] Ferrari A., Fiorino E., Giudici M., Gilardi F., Galmozzi A., Mitro N., Cermenati G., Godio C., Caruso D., de Fabiani E. (2012). Linking epigenetics to lipid metabolism: Focus on histone deacetylases. Mol. Membr. Biol..

[111] El-Osta A., Brasacchio D., Yao D., Pocai A., Jones P.L., Roeder R.G., Cooper M.E., Brownlee M. (2008). Transient high glucose causes persistent epigenetic changes and altered gene expression during subsequent normoglycemia. J. Exp. Med..

[112] Mali P., Chou B.K., Yen J., Ye Z., Zou J., Dowey S., Brodsky R.A., Ohm J.E., Yu W., Baylin S.B. (2010). Butyrate greatly enhances derivation of human induced pluripotent stem cells by promoting epigenetic remodeling and the expression of pluripotency-associated genes. Stem Cells.

[113] Martin S.L., Hardy T.M., Tollefsbol T.O. (2013). Medicinal chemistry of the epigenetic diet and caloric restriction. Curr. Med. Chem..

[114] Qiu X., Brown K.V., Moran Y., Chen D. (2010). Sirtuin regulation in calorie restriction. Biochim. Biophys. Acta.

[115] Ganguly A., Chen Y., Shin B.C., Devaskar S.U. (2014). Prenatal caloric restriction enhances DNA methylation and MeCP2 recruitment with reduced murine placental glucose transporter isoform 3 expression. J. Nutr. Biochem..

[116] Plagemann A., Harder T., Brunn M., Harder A., Roepke K., Wittrock-Staar M., Ziska T., Schellong K., Rodekamp E., Melchior K. (2009). Hypothalamic proopiomelanocortin promoter methylation becomes altered by early overfeeding: An epigenetic model of obesity and the metabolic syndrome. J. Physiol..

[117] Cho C.E., Sanchez-Hernandez D., Reza-Lopez S.A., Huot P.S., Kim Y.I., Anderson G.H. (2013). High folate gestational and post-weaning diets alter hypothalamic feeding pathways by DNA methylation in Wistar rat offspring. Epigenetics.

[118] Ly A., Hoyt L., Crowell J., Kim Y.I. (2012). Folate and DNA methylation. Antioxid. Redox Signal..

[119] Crider K.S., Yang T.P., Berry R.J., Bailey L.B. (2012). Folate and DNA methylation: A review of molecular mechanisms and the evidence for folate’s role. Adv. Nutr..

[120] Sinclair K.D., Allegrucci C., Singh R., Gardner D.S., Sebastian S., Bispham J., Thurston A., Huntley J.F., Rees W.D., Maloney C.A. (2007). DNA methylation, insulin resistance, and blood pressure in offspring determined by maternal periconceptional B vitamin and methionine status. Proc. Natl. Acad. Sci. USA.

[121] Zhou H.R., Zhang F.F., Ma Z.Y., Huang H.W., Jiang L., Cai T., Zhu J.K., Zhang C., He X.J. (2013). Folate polyglutamylation is involved in chromatin silencing by maintaining global DNA methylation and histone H3K9 dimethylation in *Arabidopsis*. Plant Cell.

[122] Xue J., Zempleni J. (2013). Epigenetic synergies between biotin and folate in the regulation of pro-inflammatory cytokines and repeats. Scand. J. Immunol..

[123] Pogribny I.P., Ross S.A., Tryndyak V.P., Pogribna M., Poirier L.A., Karpinets T.V. (2006). Histone H3 lysine 9 and H4 lysine 20 trimethylation and the expression of Suv4-20h2 and Suv-39h1 histone methyltransferases in hepatocarcinogenesis induced by methyl deficiency in rats. Carcinogenesis.

[124] Tryndyak V.P., Ross S.A., Beland F.A., Pogribny I.P. (2009). Down-regulation of the microRNAs miR-34a, miR-127, and miR-200b in rat liver during hepatocarcinogenesis induced by a methyl-deficient diet. Mol. Carcinog..

[125] Pogribny I.P., Starlard-Davenport A., Tryndyak V.P., Han T., Ross S.A., Rusyn I., Beland F.A. (2010). Difference in expression of hepatic microRNAs miR-29c, miR-34a, miR-155, and miR-200b is associated with strain-specific susceptibility to dietary nonalcoholic steatohepatitis in mice. Lab. Invest..

[126] Minor E.A., Court B.L., Young J.I., Wang G. (2013). Ascorbate induces ten-eleven translocation (Tet) methylcytosine dioxygenase-mediated generation of 5-hydroxymethylcytosine. J. Biol. Chem..

[127] Moison C., Senamaud-Beaufort C., Fourriere L., Champion C., Ceccaldi A., Lacomme S., Daunay A., Tost J., Arimondo P.B., Guieysse-Peugeot A.L. (2013). DNA methylation associated with polycomb repression in retinoic acid receptor beta silencing. FASEB J..

[128] Kuriyama M., Udagawa A., Yoshimoto S., Ichinose M., Sato K., Yamazaki K., Matsuno Y., Shiota K., Mori C. (2008). DNA methylation changes during cleft palate formation induced by retinoic acid in mice. Cleft Palate Craniofac. J..

[129] Lee E.R., Murdoch F.E., Fritsch M.K. (2007). High histone acetylation and decreased polycomb repressive complex 2 member levels regulate gene specific transcriptional changes during early embryonic stem cell differentiation induced by retinoic acid. Stem Cells.

[130] Urvalek A.M., Gudas L.J. (2014). Retinoic acid and histone deacetylases regulate epigenetic changes in embryonic stem cells. J. Biol. Chem..

[131] Urvalek A., Laursen K.B., Gudas L.J. (2014). The roles of retinoic acid and retinoic acid receptors in inducing epigenetic changes. Subcell. Biochem..

[132] Schenk T., Chen W.C., Gollner S., Howell L., Jin L., Hebestreit K., Klein H.U., Popescu A.C., Burnett A., Mills K. (2012). Inhibition of the lsd1 (KDM1A) demethylase reactivates the all-trans-retinoic acid differentiation pathway in acute myeloid leukemia. Nat. Med..

[133] Das S., Foley N., Bryan K., Watters K.M., Bray I., Murphy D.M., Buckley P.G., Stallings R.L. (2010). MicroRNA mediates DNA demethylation events triggered by retinoic acid during neuroblastoma cell differentiation. Cancer Res..

[134] Hassan Y.I., Zempleni J. (2006). Epigenetic regulation of chromatin structure and gene function by biotin. J. Nutr..

[135] Huang Y., Khor T.O., Shu L., Saw C.L., Wu T.Y., Suh N., Yang C.S., Kong A.N. (2012). A gamma-tocopherol-rich mixture of tocopherols maintains Nrf2 expression in prostate tumors of TRAMP mice via epigenetic inhibition of CpG methylation. J. Nutr..

[136] Cornwell D.G., Ma J. (2007). Studies in vitamin E: Biochemistry and molecular biology of tocopherol quinones. Vitam. Horm..

[137] Bobrowska-Korczak B., Skrajnowska D., Tokarz A. (2013). Effect of zinc and copper supplementation on the prognostic value of urinary 5-methyl-2’-deoxycytidine in DMBA-induced carcinogenesis in rats. Cancer Biomark..

[138] Wang L., Bammler T.K., Beyer R.P., Gallagher E.P. (2013). Copper-induced deregulation of microRNA expression in the zebrafish olfactory system. Environ. Sci. Technol..

[139] Yang G., Zhu Y., Dong X., Duan Z., Niu X., Wei J. (2014). TLT2-ICAM1-Gadd45alpha axis mediates the epigenetic effect of selenium on DNA methylation and gene expression in Keshan disease. Biol. Trace Elem. Res..

[140] Metes-Kosik N., Luptak I., Dibello P.M., Handy D.E., Tang S.S., Zhi H., Qin F., Jacobsen D.W., Loscalzo J., Joseph J. (2012). Both selenium deficiency and modest selenium supplementation lead to myocardial fibrosis in mice via effects on redox-methylation balance. Mol. Nutr. Food Res..

[141] Szarc vel Szic K., Ndlovu M.N., Haegeman G., Vanden Berghe W. (2010). Nature or nurture: Let food be your epigenetic medicine in chronic inflammatory disorders. Biochem. Pharmacol..

[142] Khan S.I., Aumsuwan P., Khan I.A., Walker L.A., Dasmahapatra A.K. (2012). Epigenetic events associated with breast cancer and their prevention by dietary components targeting the epigenome. Chem. Res. Toxicol..

[143] Vanden Berghe W. (2012). Epigenetic impact of dietary polyphenols in cancer chemoprevention: Lifelong remodeling of our epigenomes. Pharmacol. Res..

[144] Ong T.P., Moreno F.S., Ross S.A. (2011). Targeting the epigenome with bioactive food components for cancer prevention. J. Nutrigenet. Nutr..

